# Pilomatrixoma of Left Shoulder in an Elderly Female: A Case Report

**DOI:** 10.5704/MOJ.1307.005

**Published:** 2013-07

**Authors:** LH Tan, CL Ooi, HS Chua, O Zulkiflee

**Affiliations:** Department of Orthopaedics, Pulau Pinang Hospital, Georgetown, Malaysia; Department of Orthopaedics, Pulau Pinang Hospital, Georgetown, Malaysia; Department of Orthopaedics, Pulau Pinang Hospital, Georgetown, Malaysia; Department of Orthopaedics, Pulau Pinang Hospital, Georgetown, Malaysia

## Abstract

We report a huge pilomatrixoma of the left shoulder in an elderly female, raising fear of malignancy. Magnetic Resonance Imaging delineated the swelling and wide surgical excision was performed. Post operative recovery was uneventful. Long term follow up is necessary in view of the possibility of recurrence and malignant transformation.

**Key Words:**

Pilomatrixoma, Pilomatricoma, Calcifying Epithelioma of Malherbe, Shoulder, Upper Limb, Elderly, Female

## Introduction

Pilomatrixoma (calcifying epithelioma of Malherbe) is a benign tumour of hair follicular matrix cells based on histochemical and electron microscopic studies^1^. It was first described by Malherbe and Chenantais in 1880 as a calcified tumour from the sebaceous glands^2^. It commonly affecs children and adolescents; however, there is a smaller bell graph in the elderly^3^. It is slightly more common in females. Its occurrence in upper limb is rare and may mimic other cutaneous lesion ranging from benign to malignant tumours, at its different stages of presentation^4^.

## Case Report

A 69-year-old lady presented with left shoulder swelling for past 20 years. The swelling was gradually enlarging and associated with pain in the last two years. One month prior to consultation, the swelling ulcerated with seropurulent discharge. She had no fever nor constitutional sign and symptom of malignancy. Clinical examination revealed an 8cm x 5cm x 3cm firm exophytic swelling with central ulceration over the left deltoid region (Figure 1a). It appeared not adherent to underlying muscle. Range of motion of left shoulder was full. No cervical or axillary lymph nodes were palpable. Neurovascular status of left upper limb was normal. Plain radiograph revealed a heterogeneous soft tissue lesion with foci of opacity. Magnetic Resonance Imaging (Figure 1b) showed a well defined soft tissue mass involving the cutaneous and subcutaneous layers. It displayed iso/hypointense T1 signal to muscles, heterogeneous T2 signal and heterogeneous enhancement in post intravenous gadolinium study. Medially, there was no clear demarcation with the deltoid fascia, suggestive of fascia involvement, but the deltoid muscle appeared not involved. The radiological differential diagnoses were dermatofibrosarcoma protuberans, squamous cell carcinoma, malignant fibrous histiocytoma or nodular fasciitis.

Wide surgical excision of the left shoulder tumour was performed and wound was closed primarily (Figure 2a). Histopathological examination of the excised tumour confirmed the diagnosis of pilomatrixoma, with no evidence of malignancy (Figure 2b). On follow up review of the patient at three months, the wound had healed without complication; there was no early local recurrence and left shoulder range of motion was full. On follow up at six months, one year and one and a half years, patient showed no local recurrence of the tumour and scar was clinically quiescent, with full range of motion of the left shoulder and motor power comparable to right shoulder (Figure 3). She was able to perform daily activities independently including driving.

## Discussion

The presentation of this lady with a rapidly enlarging swelling and ulceration with seropurulent discharge associated with pain, foci calcification on plain radiograph, as well as heterogenous signal on MRI alerted on the high possibility of a malignant tumour. The clinical appearance mimicked the malignant picture of squamous cell carcinoma. Hence, without pre- excision cytological examination or intralesional biopsy, wide surgical excision of the tumour was performed. This more extensive surgical approach was performed as if it is a malignant tumour. Adjuvant chemotherapy or radiotherapy might be indicated if malignancy was confirmed.

The histopathological and microscopic examination however, showed a benign partly ulcerated circumscribed lesion formed by solid, irregular islands of epithelial cells which were embedded within a fibrocollagenous stroma. The epithelial cells were of two basic types, with the predominant cell type comprising of eosinophilic shadow cells (Ghost cell) with distinct cytoplasmic borders and a central unstained area due to lost nucleus (Figure 2b). The second cell type was composed of mostly squamous cells, with occasional basophilic cells resembling hair matrix cells. Mitotic activity was present focally but no cytological atypia was observed. The attached stroma showed foreign-body giant cell reaction and infiltration by mainly chronic mononuclear inflammatory cells. The typical ‘Ghost Cell’ appearance is suggestive of pilomatrixoma^4^.

The clinical picture of the ulcerating lesion suggested a possible suspicion of a malignant tumour and a wide surgical excision was therefore carried out promptly. A fine needle aspiration for cytological examination or intralesional biopsy (in this reported case, intralesional would have been preferred from fine needle aspiration in view of its clinical appearance) would be most helpful to reach a diagnosis and assist in landscaping the excision margin, apart from the radiological findings. A malignant lesion could have been ruled out and a marginal surgical excision would have been adequate^4^,^5^.

The surgical excision of the lesion for this case has brought about the cessation of a ulcerating lesion and its subsequent complications such as superimposed infection. The complaint of pain was being solved with the lesion being excised and the cosmesis of the shoulder preserved. The lesion may be left untreated if it is at early stage without producing significant impairment functionally or cosmetically (ie, ulceration, pain or enlarging). Although the reported local recurrence rate after complete surgical excision is rare^5^, the patient should be followed up for rapid or local recurrence as it can be a red flag for malignant transformation (pilomatrix carcinoma).

**Fig. 1a:**
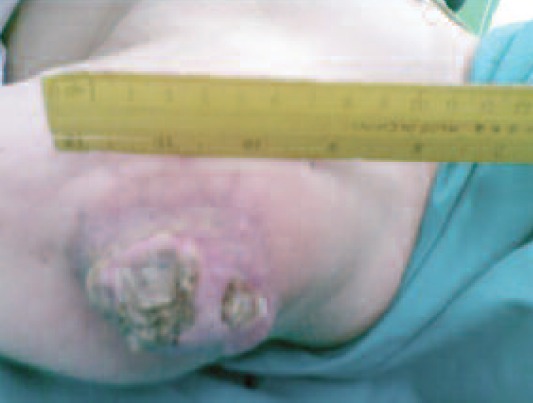
: Ulcerating pilomatrixoma left shoulder.

**Fig. 1b:**
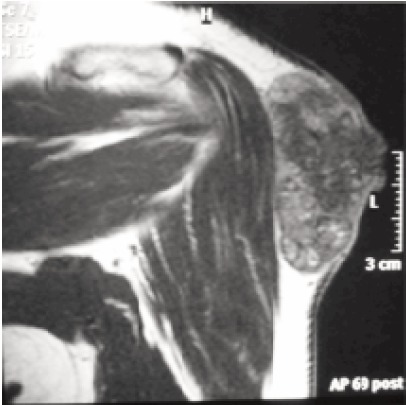
: MRI of left shoulder, coronal view shows well defined soft tissue mass involving the cutaneous and subcutaneous, with fascia involvement.

**Fig. 2a:**
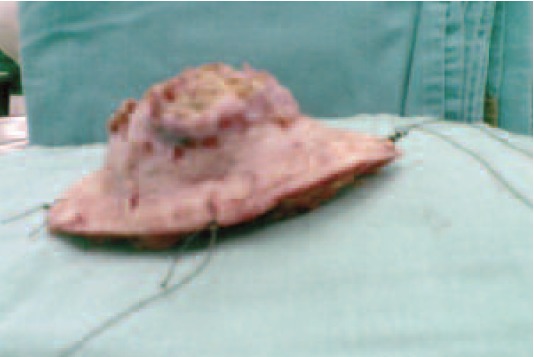
: Excised tumour.

**Fig. 2b:**
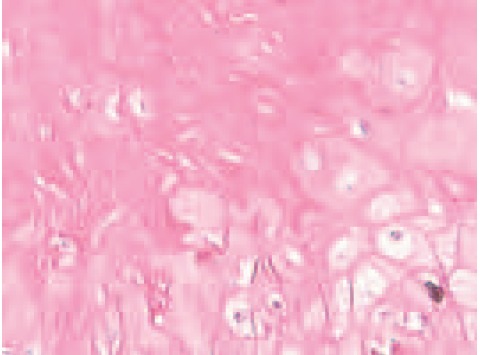
: High power magnification of tumour showing shadow/ghost cell appearance (anucleus with distinct cytoplasmic border cell).

**Fig. 3:**
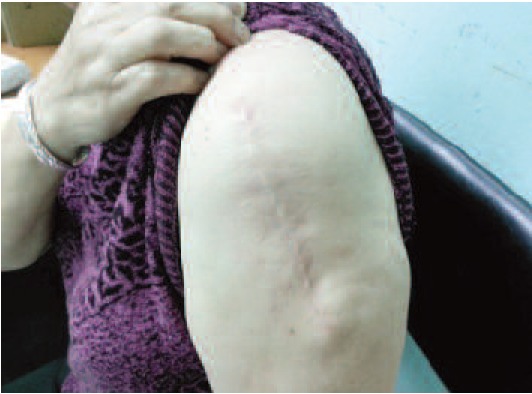
: One and half years post tumour excision.
